# Downregulation of ATOH8 induced by EBV-encoded LMP1 contributes to the malignant phenotype of nasopharyngeal carcinoma

**DOI:** 10.18632/oncotarget.8503

**Published:** 2016-03-31

**Authors:** Zifeng Wang, Jiajun Xie, Min Yan, Jing Wang, Xi Wang, Jialiang Zhang, Yan Zhang, Pengfei Li, Xinxing Lei, Qitao Huang, Suxia Lin, Xiang Guo, Quentin Liu

**Affiliations:** ^1^ Sun Yat-sen University Cancer Center, State Key Laboratory of Oncology in South China, Collaborative Innovation Center of Cancer Medicine, Guangzhou, China; ^2^ Institute of Cancer Stem Cell, Dalian Medical University, Dalian, China; ^3^ Yale Stem Cell Center, Department of Genetics, Yale University, New Haven, CT, USA

**Keywords:** nasopharyngeal carcinoma, LMP1, ATOH8

## Abstract

Mechanism for the malignant phenotype of nasopharyngeal carcinoma (NPC) remains poorly understood. Epstein-Barr virus (EBV) consistently appears in nearly all malignant NPC patient samples, suggesting the strong etiological link between the malignant phenotype and EBV infection. Here we found that the EBV-encoded latent membrane protein (LMP1) enhanced cell growth, motility, invasion and xenograft tumor growth of NPC. RNA-seq profiling analysis of LMP1-positive NPC patient tissues indicated that widespread gene repression contributed to malignant phenotype of NPC. The transcription factor binding site (TFBS) enrichment analysis indicated a subset of transcription factors including ATOH8, a novel transcript factor which belongs to the basic helix-loop-helix (bHLH) gene family inversely enriched in promoters of up-regulated genes and down-regulated genes. Importantly, the expression of ATOH8 was suppressed in both immortalized normal nasopharyngeal epithelial cells (NPEC) and NPC cells with LMP1 overexpression. The Real-Time PCR and Western Blot assays indicated that ATOH8 decreased expression in NPC cell lines and patient samples. Moreover, by gain- or loss-of-function assays, we demonstrated that ATOH8 inhibition promoted malignant phenotype, whereas ATOH8 restoration reversed malignant phenotype of NPC. Finally, we demonstrated that LMP1 inhibited ATOH8 expression by epigenetically impairing the occupancy of activating H3K4me3 and enhancing the occupancy of repressive H3K27me3 on ATOH8 promoter. Collectively, our study uncovered the occurrence of malignant phenotype of NPC induced by EBV infection and characterized a novel bHLH transcription factor ATOH8 as a new downstream target of LMP1.

## INTRODUCTION

Epstein-Barr virus (EBV) is a ubiquitous human herpesvirus. EBV infection is commonly associated with multiple human malignancies including nasopharyngeal carcinoma (NPC), Burkitt's lymphoma, Hodgkin's lymphoma, post-transplant lymphoproliferative disease, some NK/T-cell lymphomas, and a proportion of gastric carcinomas [1, 2]. It has been reported that EBV is detected consistently in nearly all NPC patient tissues [3]. EBV-associated NPC is distinctive among human solid tumors in its highly metastatic character to cervical lymph nodes and poor prognosis [4, 5]. As a key effector in EBV-driven B cell transformation and an established “transforming” gene, latent membrane protein (LMP1) displays oncogenic properties in rodent fibroblasts and induces profound morphological and phenotypic effects in epithelial cells [6]. Previous studies have indicated that NPC with high levels of LMP1 tend to be more malignant [7–11]. At present, the molecular mechanism of LMP1 in NPC tumorigenesis is largely unknown.

Atonal homolog 8 (ATOH8) is a novel transcript factor belonging to the bHLH gene family. ATOH8 is involved in the regulation of multiple developmental processes, including nervous system development, kidney development, pancreas development, and muscle development [12, 13]. In cancer studies, ATOH8 was first proposed as an oncogene in glioblastoma multiform only because of the copy number amplifications [14]. However, the following study demonstrated that the expression of ATOH8 was significantly increased 3 fold after retinoic acid (RA) administration in GBM-derived stem-like tumor-initiating cells, suggesting the different function [15]. Recently, ATOH8 was identified as a downstream effector of IL6-STAT3 signaling that compromised long-term surviving in breast cancer [16]. However, another study released alongside showed that depletion of ATOH8 reprogramed non-cancer stem cells into cancer stem cells by directly releasing AFP, CD133, OCT4 and NANOG [17, 18].

Here we found that EBV-encoded LMP1 enhanced cell growth, motility, invasion and xenograft tumor growth of NPC. Most importantly, by *in silico* analysis and gain- or loss-of function assays, we identified ATOH8 as a new downstream target of LMP1. We found ATOH8 inhibition correlated with mesenchymal status and contributes to the malignant phenotype of nasopharyngeal carcinoma.

## RESULTS

### LMP1 induces malignant phenotype of NPC cells

**Figure 1:**
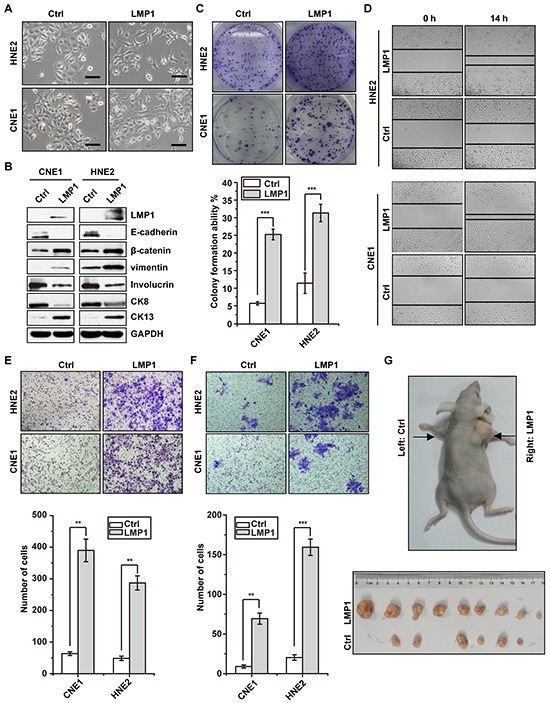
LMP1 induces malignant phenotype of NPC cells **A.** morphologic changes after induced expression of LMP1 with doxycycline in CNE1 and HNE2 cells harboring LMP1 (LMP1) or empty vector (Ctrl), respectively. Scale bars, 50 um. **B.** western blot analysis showed expression of epithelial markers E-cadherin, Involucrin, CK8, and mesenchymal markers β-catenin, vimentin and CK13 after induced expression of LMP1 with doxycycline in CNE1 and HNE2 cells harboring LMP1 or empty vector, respectively. **C.** colony formation assays showed that induced expression of LMP1 enhanced the cell growth of CNE1 and HNE2 cells. **D. & E. & F.** the wounding healing assays, transwell migration and invasion assays showed that induced expression of LMP1 enhanced the migration and invasion ability of CNE1 and HNE2 cells. **G.** mouse xenograft assay indicated that induced expression of LMP1 enhanced cell growth *in vivo*. **p < 0.01, ***p < 0.001, two-tailed Student's t-test.

To explore whether LMP1 enhance malignant phenotype of NPC cells, we stably expressed LMP1 in LMP1-negative epithelial-like CNE1 and HNE2 cells and then evaluated malignant phenotype of these cells. As shown in Figure 1A, CNE1 morphologically changed from an epithelial to a fibroblast-like, spindle-shape morphology, which indicated the phenotype transformation from epithelial status to mesenchymal status. Consistent with these morphological changes, E-cadherin was significantly suppressed, and β-catenin and vimentin were significantly activated (Figure 1B). In addition, the expression of well-differentiation markers Involucrin and CK8 were decreased, whereas the expression of poor-differentiation marker CK13 was increased (Figure 1B). Colony formation assays showed that expression of LMP1 significantly increased cell proliferation in both CNE1 and HNE2 cells (Figure 1C). Furthermore, the migration and invasion ability of both CNE1 and HNE2 cells were significantly increased along with LMP1 expression (Figure 1D, 1E & 1F). Given the phenotypic and functional changes observed *in vitro*, we next tested the function of LMP1 *in vivo* using a xenograft tumor model. As shown in Figure 1G, the size of tumor mass derived from LMP1 overexpressed cells were significantly larger than that derived from control cells. Taken together, these results suggest that LMP1 promotes tumorigenicity of NPC cells.

### Widespread gene repression in LMP1 positive tumor tissues contributes to malignant phenotype

**Figure 2:**
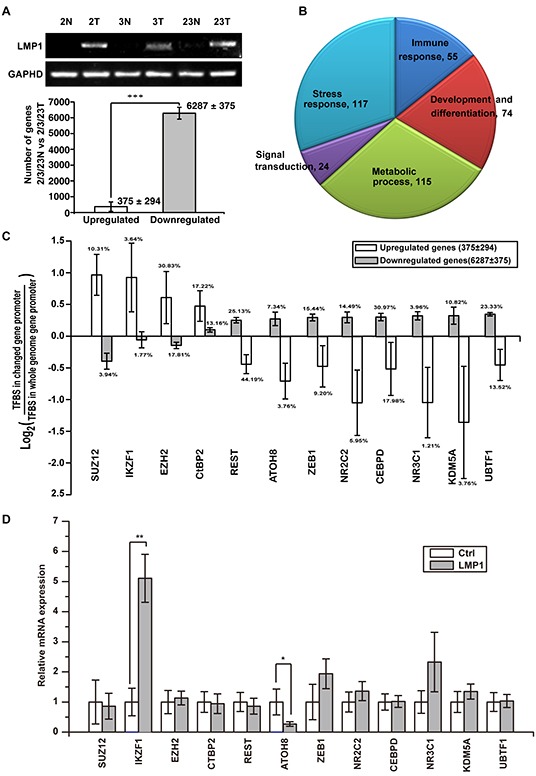
Widespread gene repression in LMP1-positive NPC contributes to tumorigenicity **A.** six RNA libraries from three paired primary NPC tumors (2T, 3T, 23T) as well as adjacent non-tumor (2N, 3N, 23N) tissues were analysed by unbiased RNA-seq assays. 6287 genes were down-regulated and 375 genes were up-regulated in all three NPC patient samples compared with paired adjacent nontumor tissues. **B.** DAVID Gene Ontology (GO) analysis of the dysregulated genes match to the functions of development and differentiation, immune responses, stress responses, signal transduction and metabolic process. **C.** transcription factor binding site (TFBS) enrichment analysis found a subset of TFBSs inversely enriched in up-regulated genes and down-regulated genes. **D.** the expression of the enriched transcription factors were examined in EBV negative NPEC or NPC cell lines NPEC1, NPEC2, CNE1 and HNE2 with or without overexpressed LMP1. *p < 0.05, **p < 0.01, ***p < 0.001, two-tailed Student's t-test.

Previous studies have shown that LMP1 activate a subset of signaling pathways such as NF-κB, JNK/SAPK, PI3K/Akt, ERK-MAPK, PLC/PKC and JAK/STAT, which activate the expression of numerous downstream effectors that enhance a variety of cellular processes such as proliferation, survival, motility and invasion [6]. To identify the genes essential for malignant phenotype of NPC cells, we sequenced six RNA libraries from three pairs of NPC tumor (2T, 3T, 23T) and adjacent non-tumor (2N, 3N, 23N) tissues as previous report [19]. LMP1 was detectable in all the tumor tissues, whereas it could not be detected in all the adjacent non-tumor tissues (Figure 2A, upper panel). All genes showing a twofold or greater up-regulation or down-regulation in the tumor tissues were chosen for further analysis. On average, 6287 genes decreased expression and only 375 genes increased expression (Figure 2A, lower panel), suggesting the correlation between the widespread gene repression and LMP1 expression. The Gene Ontology (GO) Enrichment Analysis indicated that these dys-regulated genes match to the functions of development and differentiation, immune response, stress response, signal transduction and metabolic process (Figure 2B).

To explore the core factor(s) of transcription-regulatory network modulating these dysregulated genes, we performed transcription factor binding site (TFBS) enrichment analysis. A subset of transcription factors was inversely enriched in promoters of up-regulated genes and down-regulated genes (Figure 2C). To characterize the candidate transcription factors regulated by LMP1, we transiently expressed LMP1 in four EBV negative immortalized NPEC or NPC cell lines NPEC1, NPEC2, CNE1 and HNE2. The expressions of all these transcription factors were detected by Real-Time PCR assay. The results showed that IKZF1 mRNA level was consistently up-regulated, whereas ATOH8 mRNA level was consistently down-regulated in all the four cell lines (Figure 2D).

### ATOH8 is suppressed by LMP1 and correlates with mesenchymal status of NPC

**Figure 3:**
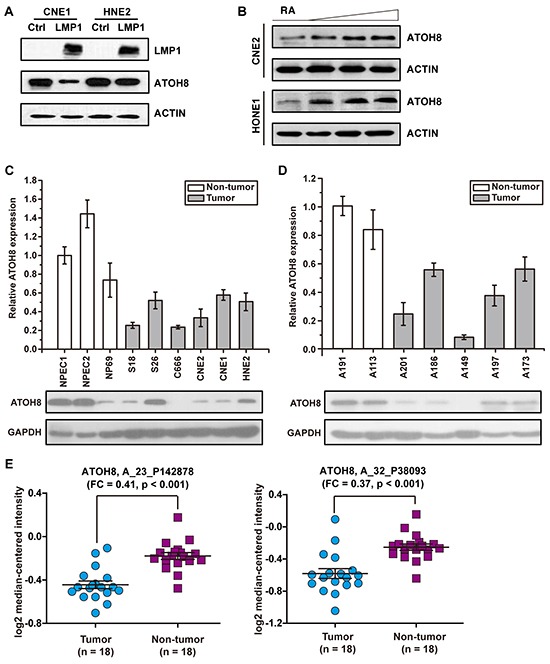
ATOH8 decreased expression in NPC **A.** western blot assays indicated that ATOH8 was down-regulated by stably overexpressed LMP1 in CNE1 and HNE2 cells. **B.** the expression of ATOH8 was increased in a dose-dependent manner in CNE2 and HONE1 cells by treating with increasing doses of RA for 48 h. **C.** the expression of ATOH8 in NPEC and NPC cell lines. NPEC1, NPEC2 and NP69 are immortalized normal human nasopharyngeal epithelial cell line. **D.** the expression of the ATOH8 in non-tumor nasopharyngeal epithelial tissues and tumor nasopharyngeal epithelial tissues. A191 and A113 are non-tumor nasopharyngeal epithelial tissues, A201, A186, A149, A197 and A173 are tumor nasopharyngeal epithelial tissues. **E.** the expression of ATOH8 in nasopharyngeal carcinoma primary tumor tissues and non-cancerous nasopharyngeal tissues according to GEO Profile (GSE53819). A_23_P142878 and A_32_P38093 are probes to detect ATOH8. FC, fold change.

The suppressive effect of LMP1 on ATOH8 was further confirmed by Western Blot assays *via* stably expressed LMP1 in CNE1 and HNE2 cells (Figure 3A). To further confirm that the expression level of ATOH8 was correlated with the EMT status of NPC, we determined the expression of LMP1, ATOH8, and EMT markers E-cadherin, vimentin, β-catenin, Twist1, Snail, Slug and ZEB1 using Real-Time PCR in 41 NPC clinical samples collecting from Sun Yat-sen University Cancer Center. Among them, 4 samples are LMP1 negative. Thus we analyzed the correlations of the expression of these genes in the remaining 37 LMP1 positive samples. The expression of LMP1, ATOH8, E-cadherin, vimentin, β-catenin, Twist1, Snail, Slug and ZEB1 were normalized to GAPDH as 2^ΔCt^. Pearson's correlation was employed using SPSS 16.0 software. As shown in Supplementary Table S1, negative correlations were observed between the expression of ATOH8 and LMP1 (Pearson = −0.425, p < 0.01), ATOH8 and vimentin (Pearson = −0.334, p < 0.05), ATOH8 and β-catenin (Pearson = −0.370, p < 0.05), ATOH8 and Slug (Pearson = −0.458, p < 0.01), respectively. A positive correlation was observed between the expression of ATOH8 and E-cadherin (Pearson = 0.456, p < 0.01). We further induced differentiation of two poor-differentiated cell lines CNE2 and HONE1 by using RA as previously shown [19]. The result showed that the expression of ATOH8 was increased in a dose-dependent manner in CNE2 and HNOE1 cells by treating with increasing doses of RA for 48 h (Figure 3B).

We further detected the expression of ATOH8 mRNA level and protein level in NPEC cell lines, NPC cell lines, non-tumor nasopharyngeal epithelial tissues and tumor nasopharyngeal epithelial tissues. As shown in Figure 3C&3D, the results of Real-Time PCR assays and Western Blot assays indicated that ATOH8 significantly decreased expression in NPC cells or patient tissues as compared with the non-tumor cells or tissues. Moreover, to strengthen the result, we analyzed ATOH8 expression in GEO database accessing number GSE53819. The dataset presents gene expression profiles of 18 nasopharyngeal carcinoma primary tumor tissues and 18 non-cancerous nasopharyngeal tissues. Two probes named A_23_P142878 and A_32_P38093 were used to detect ATOH8 expression. As shown in Figure 3E, both probes indicated that ATOH8 significantly decreased expression in NPC patient tissues. Collectively, these data demonstrated that ATOH8 decreased expression in NPC and suggested that suppression of ATOH8 was related to the mesenchymal status of NPC cells.

### ATOH8 inhibition enhances malignant phenotype of NPC

**Figure 4:**
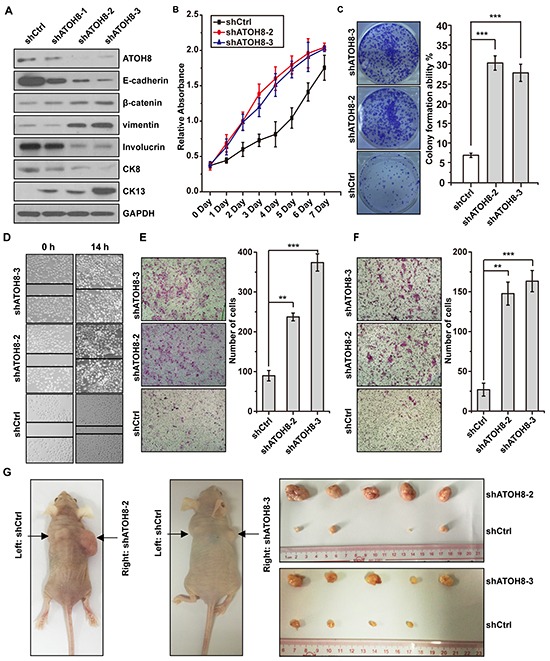
ATOH8 inhibition enhances malignant phenotype of NPC **A.** western blot analysis with indicated antibodies in CNE1 cells harboring ATOH8 interfering RNA (shATOH8-1, shATOH8-2, shATOH8-3), or negative control RNA (shCtrl) after induced with doxycycline. **B.** MTT assays showed that ATOH8 inhibition enhanced cell proliferation of CNE1 cells. **C.** Colony formation assays showed that ATOH8 inhibition enhanced the cell growth of CNE1 cells. **D. & E. & F.** the wounding healing assays, transwell migration and invasion assays showed that ATOH8 inhibition enhanced the migration and invasion ability of CNE1 cells. **G.** mouse xenograft assays indicated that ATOH8 inhibition enhanced CNE1 cell growth *in vivo*. **p < 0.01, ***p < 0.001, two-tailed Student's t-test.

To examine whether ATOH8 inhibition directly enhance malignant phenotype of NPC cells, we first silenced ATOH8 expression in the epithelial-like CNE1 cells. Knock down efficiency was analyzed by Western blot assays (Figure 4A). ATOH8 inhibition decreased the expression of epithelial marker E-cadherin and increased the expression of mesenchymal markers β-catenin and vimentin (Figure 4A). In addition, the expression of well-differentiation markers Involucrin and CK8 were decreased, whereas the expression of poor-differentiation marker CK13 was increased (Figure 4A). 3-(4,5-dimethylthiazol-2-yl)-2,5-diphenyltetrazolium bromide (MTT) proliferation assays and colony formation assays indicated that ATOH8 inhibition accelerated cell proliferation and colony formation in CNE1 cells (Figure 4B & 4C). Transwell migration assays and matrigel invasion assays demonstrated that ATOH8 inhibition significantly enhanced migration and invasion ability of CNE1 cells (Figure 3D, 3E & 3F). The xenograft tumor model assays indicated the size of tumor mass derived from ATOH8 depletion cells were obviously larger than that derived from control cells. Taken together, these results demonstrated that ATOH8 inhibition enhanced malignant phenotype of NPC cells.

### ATOH8 restoration reverses malignant phenotype of NPC

**Figure 5:**
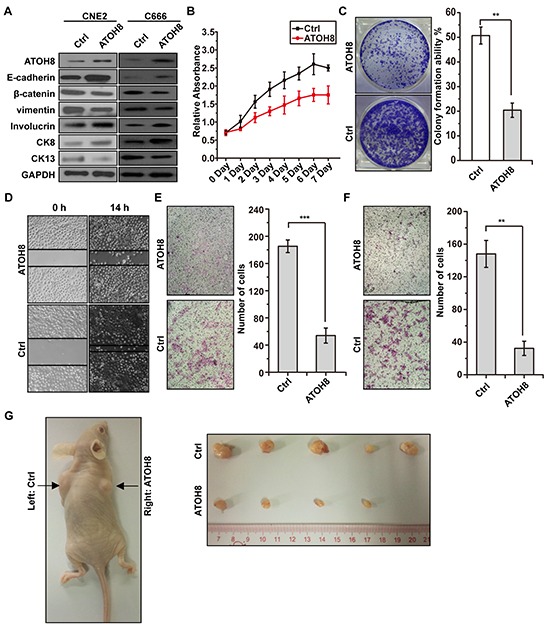
ATOH8 restoration reverses malignant phenotype of NPC **A.** western blot analysis with indicated antibodies in CNE2 and C666 cells with or without ATOH8 overexpression. ATOH8 was stably overexpressed in CNE2 cells and transiently overexpressed in C666 cells. **B.** MTT assays showed that ATOH8 restoration inhibited proliferation of CNE2 cells. **C.** colony formation assays showed that ATOH8 restoration inhibited cell growth of CNE2 cells. **D. & E. & F.** the wounding healing assays, transwell migration and invasion assays showed that ATOH8 restoration inhibited the migration and invasion ability of CNE2 cells. **G.** mouse xenograft assay indicated that ATOH8 restoration inhibited CNE2 cell growth *in vivo*. **p < 0.01, ***p < 0.001, two-tailed Student's t-test.

To explore whether restoration of ATOH8 expression reverse malignant phenotype of NPC cells, we stably introduced wild-type ATOH8 into mesenchymal-like EBV-negative CNE2 cells and transiently introduced wild-type ATOH8 into mesenchymal-like EBV-positive C666 cells. As shown in Figure 5A, ATOH8 restoration increased the expression of epithelial markers E-cadherin, decreased the expression of mesenchymal markers β-catenin and vimentin in both cells. In addition, the expression of well-differentiation markers Involucrin and CK8 were increased, whereas the expression of poor-differentiation marker CK13 was decreased in both cells. Functional studies demonstrated that ATOH8 restoration significantly inhibited cell proliferation, colony formation, migration and invasion of CNE2 cells (Figure 5B & 5C & 5E & 5F). The xenograft tumor model assays indicated that ATOH8 restoration in CNE2 cells significantly inhibited the growth of the tumor mass (Figure 5G). Taken together, these results demonstrated that ATOH8 restoration reverses malignant phenotype of NPC.

### ATOH8 is epigenetically silenced in NPC cells induced by LMP1

**Figure 6:**
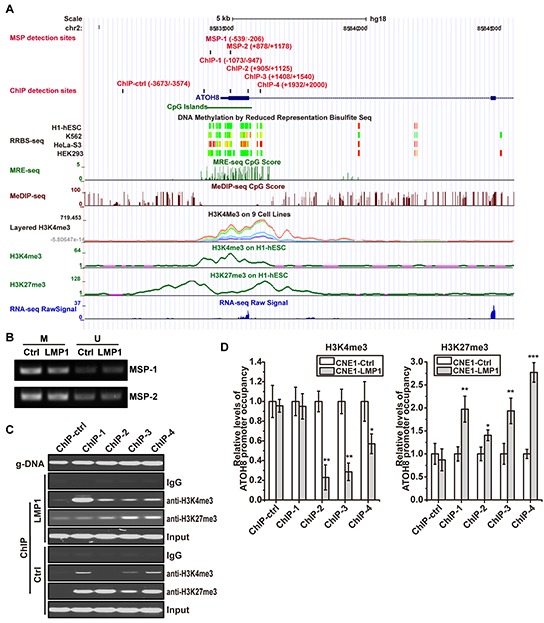
ATOH8 is epigenetically silenced in NPC cells induced by LMP1 **A.** the promoter characteristics of ATOH8 according to UCSC genome browser. RRBS, MER-seq CpG and MeDIP CpG tracks present the methylation status of ATOH8 promoter in different cells. Layered H3K4me3, H3K4me3 and H3K27me3 tracks display chromatin states of ATOH8 promoter. MSP-1 and MSP-2 indicated methylation specific PCR (MSP) detection regions. ChIP-ctrl, ChIP-1, ChIP-2, ChIP-3 and ChIP-4 indicated ChIP Real-Time PCR detection regions. **B.** products of MSP with indicated regions in ATOH8 promoter in CNE1 cells were analysis with agarose gel electrophoresis. M, methylated template. U, unmethylated template. **C. & D.** products of ChIP using H3K4me3 or H3K27me3 antibody in CNE1 cells were subjected to PCR and Real-Time PCR analysis. The relative levels of ATOH8 promoter occupancy were examined by Real-Time PCR and calculated using ΔΔCt value. g-DNA indicated the primers work well when using genome DNA as template. *p < 0.05, **p < 0.01, ***p < 0.001, two-tailed Student's t-test.

To characterize the mechanism that is responsible for ATOH8 suppression in NPC, we examined mRNA level of ATOH8 in both immortalized NP cells NPEC1, NPEC2 and NPC cells CNE1 and HNE2 with LMP1 overexpression. As shown in Figure 2D, LMP1 significantly decreased ATOH8 mRNA levels in all these cells, suggesting that LMP1 suppressed ATOH8 expression at transcriptional level. Thus we analyzed the promoter region of ATOH8 through UCSC genome browser with the ENCODE annotation data [20]. The DNA Methylation by Reduced Representation Bisulfite Seq (RRBS-seq) track, Methyl-sensitive restriction enzyme digest and sequencing (MRE-seq) track and Methylated DNA immunoprecipitation and sequencing (MeDIP-seq) track showed that the methylation status of ATOH8 promoter was variable in different cells (Figure 6A). However, methylation specific PCR (MSP) assay showed that there was no significant difference in methylation status of selected promoter regions MSP-1 and MSP-2 in LMP1 introduction cells and control cells (Figure 6B).

The Layered H3K4me3 track, and H3K4me3/ H3K27me3 on H1-hESC tracks indicated the variable modification of histone H3, suggesting the role of histone modification in regulation of ATOH8 promoter activity (Figure 6A). Thus we evaluated the occupancy of activating H3K4me3 and repressive H3K27me3 on ATOH8 promoter in LMP1 introduction cells and control cells. As shown in Figure 6C & 6D, LMP1 significantly impairing the occupancy of activating H3K4me3 and enhancing the occupancy of repressive H3K27me3 on ATOH8 promoter. Collectively, these data demonstrated that LMP1 epigenetically silenced ATOH8 expression by modulating the methylation modification of H3K4 and H3K27 in NPC cells.

## DISCUSSION

Growing evidence for the oncogenic property of LMP1 has been obtained from studies investigating the lymphomagenesis [21]. By activating several important cellular signaling pathways like NF-kB, JNK, JAK/STAT and PI3K pathway, LMP1 activates anti-apoptotic gene products, such as BCL2, AP-1, CD40, CD54 and also cytokines IL6 and IL8 to exhibit its oncogenic characteristics [22]. In NPC carcinogenesis, the function and mechanism of LMP1 remains speculative. Here we found that LMP1 inhibited transcription factor ATOH8 expression to endow the malignant phenotype of nasopharyngeal carcinoma.

Our study showed that LMP1 inhibited ATOH8 expression by epigenetically impairing the occupancy of activating H3K4me3 and enhancing the occupancy of repressive H3K27me3 on ATOH8 promoter. However, the precise mechanism between LMP1 and epigenetic machinery is still uncharted. KDM5A and EZH2 are epigenetic factors regulated histone H3K4 demethylation and H3K27 methylation [23, 24]. Our assay indicated that LMP1 did activate their expression in part of cell lines, but not consistently and significantly in all cell lines (Figure 2D). Hence, in our current study, we can't well explain how LMP1 changed the histone modification.

bHLH transcription factors control numerous aspects of vertebrate organ development and function [25]. Within the atonal superfamily of bHLH factors, ATOH8 is the sole mammalian member of the Net family [26]. Previous studies of ATOH8 in the mouse, zebrafish, and cultured cells have associated this transcription factor with a very broad variety of biological roles of embryonic development and vertebrate tissue-specific differentiation in the central nervous system, liver, pancreas, kidney, skeletal muscle, and eye [27–31]. Whether ATOH8 performs lineage determination function in nasopharynx development warrants further study.

In cancer studies, ATOH8 was an emerging star with ambiguity functions: it was proposed both as oncogene or tumor suppressor gene in glioblastoma, breast cancer and HCC [14–18]. By exploring Oncomine data base [32], we found that ATOH8 decreased expression in bladder cancer, breast cancer, lung cancer, ovarian cancer and prostate cancer, whereas it increased expression in brain and CNS cancer and esophageal cancer, suggesting the divergent function of ATOH8 in human cancers.

Previous studies demonstrated that the expression of ATOH8 was activated by a shear-stress-responsive in endothelial differentiation and phenotypic modulation, or IL6-STAT3 signaling in breast cancer, or inhibited by promoter methylation in part of HCC cell lines [13, 16, 17]. Comparative genomic analyses of the regulatory elements revealed a replacement of the ancestral TATA box by CpG-islands in the eutherian mammals and an evolutionary tendency for TATA box reduction in vertebrates in general [12]. The transition from a TATA box-type promoter to a CpG island-type promoter suggests the important of epigenetic regulation in ATOH8 expression and the fine-tuning of gene expression responding to minor changes in cellular environment. In our study, we tried to connect downregulation of ATOH8 with IL6-STAT3 pathway, or the DNMT1-CpG island methylation pathway, but the attempts were failed.

Epithelial-mesenchymal transition has recently been proposed as a major initiation process of metastasis in several types of invasive human malignancies. The genesis of EMT and its oncogenic role in carcinomas are beginning to be clarified [33–36]. Our study presented a correlation between EMT markers and ATOH8, suggesting the novel emerging transcription factor ATOH8 appears to be a bona fide EMT regulator that is specifically suppressed by exogenous LMP1 to promote NPC tumor metastasis.

Collectively, in the present study, we demonstrated that EBV-encoded LMP1 induced malignant phenotype of NPC cells through widespread gene repression and identified a novel transcript factor ATOH8 as an intracellular downstream target. We demonstrated that LMP1 inhibited ATOH8 expression by epigenetically impairing the occupancy of activating H3K4me3 and enhancing the occupancy of repressive H3K27me3 on ATOH8 promoter. These new data strengthens our view that EBV infection contributes to the malignant phenotype of NPC. These findings also raise the possibility that ATOH8 as the pivotal regulator in NPC tumorigenesis.

## MATERIALS AND METHODS

### Cell lines and cell culture

The EBV negative NPC cell lines CNE1, CNE2, HNE2, HONE1, S18 and S26 were obtained from Dr. Chaonan Qian (Sun Yat-sen University, Guangzhou, China). The EBV positive NPC cell line C666 and the EBV negative immortalized normal human nasopharyngeal epithelial cell lines NP69, NPEC1 and NPEC2 were obtained from professor Musheng Zeng (Sun Yat-sen University, Guangzhou, China) [37]. CNE1, CNE2, HNE2, HONE1, S18 and S26 were HPV-18 positive, while C666 was HPV-18 negative (Supplementary Table S2). The STR profiling of CNE1, HNE2 and C666 were analyzed by using the GoldeneyeTM 20A kit (Peoplespot, Beijing, China). The results were compared with the published results from independent laboratories including The University of Hong Kong (HKU), The Chinese University of Hong Kong (K-W Lo's lab) and Johns Hopkins Singapore (JHS) [38, 39]. The STR profiling of CNE1, HNE2 and C666 from different laboratories were very similar and just demonstrated little allelic variations, which should due to microsatellite instability and long-term culture (Supplementary Table S3). CNE1, CNE2, HNE2, HONE1, S18 and S26 were maintained in RPMI 1640 (Invitrogen) supplemented with 10% fetal bovine serum (FBS, Gibco). C666 was maintained in RPMI 1640 (Invitrogen) supplemented with 20% FBS (Gibco). NP69, NPEC1 and NPEC2 were maintained in keratinocyte/serum-free medium (Invitrogen). All these cells were incubated at 37°C in a humidified chamber containing 5% CO2.

### Tissue samples

All clinical specimens used for Real-Time PCR and Western Blot analysis were collected from NPC patients at Sun Yat-sen University Cancer Center. Patients’ consent and approval from Sun Yatsen University Cancer Center Institute Research Ethics Committee were obtained for the use of these clinical materials. For each sample, genomic DNA, total RNA, and total protein were simultaneously purified from the same tissue by using AllPrep DNA/RNA/Protein Mini Kit (Qiagen, Cat number 80004) according to the manufactures’ instructions.

### Plasmid constructs

LMP1 expression vector pLVX-TRE3G-LMP1-IRES-EGFP was constructed by insert LMP1 and EGFP into pLVX-TRE3G-IRES control vector. ATOH8 overexpression vector was constructed by cloning ATOH8 ORF into pLVX-DsRed-Monomer-N1 Vector. The primer pairs used are shown in Supplementary Table S4. The sequence and orientation of all inserts were confirmed by sequencing. To construct shRNA vectors for knocking down endogenous ATOH8, hairpin encoding oligonucleotides (shATOH8-1, shATOH8-2, shATOH8-3, Supplementary Table S4) were annealed and ligated into the pLKO-tet-on shRNA expression vector (Addgene). The following targeting sequence was used, shATOH8-1: AGT TCC TAC TCG TCA ATT T; shATOH8-2: TGT GCT CAA CCA TCT GCT T;and shATOH8-3: GGT GCC GTG CTA CTC ATA T. The sequence and orientation of all inserts were confirmed by sequencing.

### Transient plasmid transfection

CNE1, CEN2 and HNE2 cells were transiently transfected with indicated vectors by Lipofectamine 2000 transfection reagent (Invitrogen) in accordance with the manufacturer's instructions. C666 cells were transiently transfected with indicated vectors by FuGENE HD transfection reagent (Promega) in accordance with the manufacturer's instructions.

### Lentiviral production and transduction

The lentiviral packaging plasmid psPAX2 and envelope plasmid pMD2.G were purchased from Addgene. Production of lentiviral particles containing ATOH8 overexpression fragment or shRNA fragments and infection NPC cell lines followed a previous report [40].

### Western blot analysis and antibodies

Cells were lysed on ice in RIPA lysis buffer (Thermo) with protease inhibitor mixture (Sigma) and phosphatase inhibitor (Pierce). Protein concentration was determined by using the Bradford dye method. Equal amounts of cell extracts were subjected to electrophoresis in 8-12% gradient sodium dodecyl sulfate-polyacrylamide gel electrophoresis (SDS-PAGE) gels and then transferred to polyvinylidene difluoride (PVDF) membranes (Millipore) for antibody blotting. The following antibodies were used: LMP1 (1:500 dilution), ATOH8 (1:1,000, Abcam, ab106377), involucrin (1:1,000 dilution, Abcam, ab14505), CK8 (1:10,000 dilution, Epitomics,2032-1), CK13 (1:5,000 dilution, Epitomics, 2713-1), GAPDH (1:5,000 dilution, proteintech, 60004-1-Ig), E-cadherin (1:1,000 dilution, Cell Signaling Technology 3195), vimentin (1:2,000 dilution, Epitomics, 2707-1,). Horseradish peroxidase-conjugated goat anti-mouse or goat anti-rabbit IgG (1:5,000 dilution, Pierce, 31430 and 31460) was used as a secondary antibody. Proteins were visualized with a Super Signal West Pico chemiluminescence kit (Pierce).

### Total RNA extraction, cDNA synthesis, and Real-Time PCR

For RNA extraction, the total RNA was extracted by using TRIzol (Invitrogen), in accordance with the manufacturer's instructions. For cDNA synthesis, 3 μg of total RNA was reverse-transcribed by the SuperScriptTM III First-Strand synthesis system (Invitrogen) after DNase I treatment (Invitrogen). Quantitative Real-Time PCR was subsequently performed in triplicate with a 1:10 dilution of the resultant cDNA by using the Light-Cycler Roche 480 (Roche Molecular Systems) with the Light-Cycler Roche 480 master kit. The primer pairs used for PCR analysis are shown in Supplementary Table S4. All mRNA quantification data were normalized to GAPDH. The Ct value for each sample was calculated by the ΔΔCt method as described previously [41].

### RNA-seq and TFBS enrichment analysis

We sequenced six RNA libraries from three pairs of NPC tumor (2T, 3T, 23T) and adjacent non-tumor (2N, 3N, 23N) tissues as previous report [19]. We have deposited RNA-Seq data in the National Center for Biotechnology Information Sequence Read Archive under accession no. SRA064011. All genes showing a twofold or greater upregulation or downregulation in the tumor tissues were chosen for further Gene Ontology (GO) enrichment analysis and Transcription Factor Binding Site (TFBS) enrichment analysis. GO enrichment analysis was performed using DAVID online tools [42]. TFBS enrichment analysis was performed as following: 1. Obtaining TFBSs of transcription factors from ENCODE web site and JASPAR web site. 2. Counting TFBS in up-regulated and down-regulated genes, respectively. 3. Statistical analyzing differential transcription factor using hypergeometric test as the following formula. N, the number of TFBS binding genes in genome. n, the number of TFBS binding genes in dys-regulated genes. M, the number of single transcription factor binding genes in genome. m, the number of single transcription factor binding genes dys-regulated genes.

$$\text{P}=1-\sum_{i=0}^{m-1}\frac{\left(\begin{array}{c} M\\ i \end{array}\right)\left(\begin{array}{c} N-M\\ n-i \end{array}\right)}{\left(\begin{array}{c} N\\ n \end{array}\right)}$$

### Bisulfite treatment and promoter methylation analysis

Genomic DNA was extracted from NPC cell lines by phenol-chloroform method followed by bisulfite modification according to the manufacturer's instructions (Active Motif). Primer MSP-1-M and MSP-2-M were used for methylated reaction, while primer MSP-1-U and MSP-2-U were used for unmethylated reaction. MSP-1-M and MSP-1-U were used to detect MSP-1 region (−539/−206), while MSP-2-M and MSP-2-U were used to detect MSP-2 region (+878/+1178). Primer sequences were shown in Supplementary Table S4.

### MTT analysis of cell proliferation

Cells were seeded at a density of 1000 cells per well onto a 96-well plate with the complete medium (100 μl/well) and then were incubated at 37°C in a humidified environment with 5%CO2. At days 1-6, supernatants were removed, and the cells were incubated with 20μl of 5 mg/ml MTT reagent (Sigma) at 37°C for 4 h. At the end of incubation, MTT reagent was removed from each well and replaced by 100 μl of Dimethyl sulfoxide (DMSO, Invitrogen). The plate was gently agitated for 10 min, and the absorbance (A) at 490 nm was determined by an ELISA reader (Bio-Rad).

### Colony formation analyses

For the colony formation assay, Approximately 500-1,000 cells were seeded into six-well plate. After culture for 7 days, surviving colonies (>50 cells per colony) were counted and stained using crystal violet (Sigma-Aldrich). Triplicate independent experiments were performed.

### Wounding healing assay

Cell migration was assessed by measuring the movement of cells into a scraped; a cellular area created by a 200 μl pipette tube, and the spread of wound closure was observed after 48 and 60 hours and photographed under a microscope.

### Transwell migration assay and Matrigel invasion assay

For transwell migration assays, 1 × 10^5^ cells were added to the top non-coated chamber (24-well insert; pore size, 8μm; BD Biosciences). For Matrigel invasion assays, 2 × 10^5^ cells were plated in the top chamber with Matrigel-coated membrane (24-well insert; pore size, 8 μm; BD Biosciences). In both assays, cells were plated in RPM1640 medium without serum or growth factors, and RPM1640 medium supplemented with 10% FBS was used as a chemoattractant in the lower chamber. The cells were incubated for 18h and cells that did not migrate or invade through the pores were removed by a cotton swab. Cells on the lower surface of the membrane were fixed and stained with crystal violet (Sigma-Aldrich). The number of cells was counted in ten fields under a ×20 objective lens and imaged using SPOT imaging software (Nikon, Japan).

### Chromatin immunoprecipitation (ChIP)

ChIP assays carried out by using EZ-Magna ChIP kit (Millipore), in accordance with the manufacturer's instructions. Immunoprecipitations were performed overnight with H3K4me3 (Abcam, ab8580, ChIP Grade, 5μg), H3K27me3 (Millipore, 07-449, ChIP Grade, 5μg) or IgG (provided in kit, 1μg) antibodies. Semi-quantitative RT PCR and Real-Time PCR were performed by using the Light-Cycler Roche 480 (Roche Molecular Systems) with the Light-Cycler Roche 480 master kit. The primer pairs used for PCR analysis named ChIP-ctrl (−3673/−3574), ChIP-1 (−1073/−947), ChIP-2 (+905/+1125), ChIP-3 (+1408/+1540) and ChIP-4 (+1932/+2000) are shown in Supplementary Table S4. All data were normalized to input [43].

### Tumor xenografts in nude mice

We subcutaneously injected approximately 1 × 10^6^ to 2 × 10^7^ of cells into the right or left dorsal flank of 4 week old BALB/c athymic nude mice (nu/nu, male and female) with each experimental group consisting of 6 mice. Care of experimental animals was approved by Institutional Animal Care and Use Committee of Sun Yat-Sen University and in accordance with national guidelines for the care and maintenance of laboratory animals.

### Statistical analysis

The Microsoft Excel (Excel in Microsoft Office 2010 for Windows) was used for data analysis. Data were shown as means ±SD. Each experiment was performed in triplicate and repeated at least three times. Statistical analyses for detection of significant differences between the control and the experimental groups were carried out by using an unpaired two-tailed Student's t test (*, *p* ≤ 0.05; **, *p* ≤ 0.01; ***, *p* ≤ 0.001).

## SUPPLEMENTARY TABLES





## Abbreviations

NPC: nasopharyngeal carcinoma
EBV: Epstein-Barr virus
LMP1: latent membrane protein
EMT: epithelial–mesenchymal transition
TFBS: transcription factor binding site
bHLH: basic helix-oop-helix
ATOH8: Atonal homolog 8
GO: Gene Ontology
RA: retinoic acid
MTT: 3-(4,5-dimethylthiazol-2-yl)-2,5-diphenyltetrazolium bromide
RRBS-seq: Reduced Representation Bisulfite Sequencing
MRE-seq: Methyl-sensitive restriction enzyme digest and sequencing
MeDIP-seq: Methylated DNA immunoprecipitation and sequencing
5′Aza: 5-aza-2′-deoxycytidine
FBS: fetal bovine serum
shRNA: short-hairpin RNA
SDS-PAGE: sodium dodecyl sulfate-polyacrylamide gel electrophoresis
PVDF: polyvinylidene difluoride
DMSO: Dimethyl sulfoxide
ChIP: Chromatin Immunoprecipitation
HCC: hepatocellular carcinoma
