# Supplementary material for: Downregulation of ATOH8 induced by EBV-encoded LMP1 contributes to the malignant phenotype of nasopharyngeal carcinoma
Source: Oncotarget. 2016 Mar 31;7(18):26765–79. doi: 10.18632/oncotarget.8503 (PMC5042013; doi:10.18632/oncotarget.8503)
Supplement: Supplementary file 1 [file oncotarget-07-26765-s001.pdf]

SUPPLEMENTARY TABLES

Supplementary Table S1: The expression correlation of LMP1, ATOH8, E-cadherin, Vimentin, β-catenin, Twist1, Snail, Slug and ZEB1 in LMP1 positive NPC clinical samples

		LMP1	ATOH8	E-cadherin	Vimentin	β-catenin	Twist1	Snail	Slug	ZEB1
LMP1	Pearson Correlation	1.000	-0.425	-0.280	0.381	0.159	0.031	0.310	0.174	0.024
	Sig. (2-tailed)		0.009	0.093	0.020	0.348	0.856	0.062	0.304	0.886
	N	37	37	37	37	37	37	37	37	37
ATOH8	Pearson Correlation	**-0.425**	1.000	**0.456**	**-0.334**	**-0.370**	-0.060	-0.317	**-0.458**	0.072
	Sig. (2-tailed)	**0.009**		**0.005**	**0.043**	**0.024**	0.726	0.056	**0.004**	0.670
	N	37	37	37	37	37	37	37	37	37
E-cadherin	Pearson Correlation	-0.280	0.456	1.000	-0.015	-0.170	-0.063	-0.209	-0.300	0.399
	Sig. (2-tailed)	0.093	0.005		0.927	0.314	0.713	0.213	0.072	0.015
	N	37	37	37	37	37	37	37	37	37
Vimentin	Pearson Correlation	0.381	-0.334	-0.015	1.000	-0.048	0.242	0.273	0.158	0.012
	Sig. (2-tailed)	0.020	0.043	0.927		0.777	0.149	0.102	0.351	0.942
	N	37	37	37	37	37	37	37	37	37
β-catenin	Pearson Correlation	0.159	-0.370	-0.170	-0.048	1.000	0.003	0.089	0.375	-0.130
	Sig. (2-tailed)	0.348	0.024	0.314	0.777		0.985	0.600	0.022	0.443
	N	37	37	37	37	37	37	37	37	37
Twist1	Pearson Correlation	0.031	-0.060	-0.063	0.242	0.003	1.000	0.199	0.280	0.179
	Sig. (2-tailed)	0.856	0.726	0.713	0.149	0.985		0.238	0.094	0.290
	N	37	37	37	37	37	37	37	37	37
Snail	Pearson Correlation	0.310	-0.317	-0.209	0.273	0.089	0.199	1.000	0.436	0.083
	Sig. (2-tailed)	0.062	0.056	0.213	0.102	0.600	0.238		0.007	0.627
	N	37	37	37	37	37	37	37	37	37
Slug	Pearson Correlation	0.174	-0.458	-0.300	0.158	0.375	0.280	0.436	1.000	0.051
	Sig. (2-tailed)	0.304	0.004	0.072	0.351	0.022	0.094	0.007		0.763
	N	37	37	37	37	37	37	37	37	37
ZEB1	Pearson Correlation	0.024	0.072	0.399	0.012	-0.130	0.179	0.083	0.051	1.000
	Sig. (2-tailed)	0.886	0.670	0.015	0.942	0.443	0.290	0.627	0.763	
	N	37	37	37	37	37	37	37	37	37

Supplementary Table S2: HPV DNA in NPC cell lines

Cell line	CNE-1	CNE-2	HONE-1	SUNE-1	5-8F	6-10B	SUNE-2	C666	S18	HNE-2
HPV-18	+	+	+	+	+	+	+	-	+	+
HPV-16	-	-	-	-	-	-	-	-	-	-

Supplementary Table S3: STR profiles of CNE1, HNE2 and C666 cells from different laboratories

See Supplementary File 1

Supplementary Table S4: Primers used in this study

name	Sense primer (5' to 3')	Anti-sense primer (5' to 3')	Application
ATOH8 ORF	CGCCGAATTCACCATGG AGCACATCCCGGTCCT	GCCTGGATCCACTCC CTCCTTGCGCTTCTTGG	ATOH8 Cloning
qGAPDH	GAATCTACTGGCGTCTTCACC	GTCATGAGCCCTTCCACGATGC	Real-Time PCR for GAPDH
qATOH8	TGCCAAGAAGCGCAAGGAG	GTGAGGGCGGAGGGGAGAG	Real-Time PCR for ATOH8
qLMP1	CGGAAGAGGTTGA AAACAAAGGA	GTACCCAAAAGCAGCGTAGGAAG	Real-Time PCR for LMP1
qSUZ12	AAATCGTGAGGA TGGGGAAAAGAC	CGAAGAGTGAACTGCAACGTAGGT	Real-Time PCR for SUZ12
qIKZF1	GCCATCAACAACGCCATCAA	AGCAGCAGCAGGTTCTCCAC	Real-Time PCR for IKZF1
qEZH2	GGGGGAGAGAA CAATGATAAAGAAG	CAATTAACCTAGCAATGGCACAGAA	Real-Time PCR for EZH2
qCTBP2	AGAGCGACTGCGTCTCCTTG	TCGTATCCTGCCCTCCTTGA	Real-Time PCR for CTBP2
qREST	GTTAGAACTCAT ACAGGAGAACGCC	CCACATAACTGCACTGATCACATTT	Real-Time PCR for REST
qZEB1	TCAAATGCACTG AGTGTGGAAAAG	GATGCTGAAAGAGACGGTGAAGAA	Real-Time PCR for ZEB1
qCEBPD	CCGCCCCCGCCATGTAC	CCGCCCGCCTTGTGATT	Real-Time PCR for CEBPD
qNR3C1	ACTCTGCCTGGTGTGCTCTGAT	TGCTGTCCTTCCACTGCTCTTT	Real-Time PCR for NR3C1
qKDM5A	GTGGTGAGCAGTGTGTCATCTTCT	CTGGCTGTGTCCTTCATGTCGTAG	Real-Time PCR for KDM5A
qUBTF1	GCAAGCTCAAATGGGTGGAGAT	AAGAAGCGGAAATAAGGGGTCA	Real-Time PCR for UBTF1
qDNMT3a	TTCGCTAATAACCACGACCAGG	CATCAAAGAGAGACAGCACCCG	Real-Time PCR for DNMT3a
qDNMT3b	CCAACAACACGCAACCAGAGAA	TGCCACAAGACAAACAGCCATC	Real-Time PCR for DNMT3b
qE-cadherin	GAGAACGCATTGCCACATACACT	CCTTCCATGACAGACCCCTTAAA	Real-Time PCR for E-cadherin
qVimentin	TCAGAATATGAAGGAGGAAATGGC	TCAGGGAGGAAAAGTTTGGAAGAG	Real-Time PCR for Vimentin
qβ-catenin	GCAACTAAACAGGAAGGGATGGA	CCCGAAGGACAGTACGCACAAGA	Real-Time PCR for β-catenin
qTwist1	GCGGAAGATCATCCCCACG	CCCCCTCCATCCTCCAGAC	Real-Time PCR for Twist1
qSnail	CAGGACAGAGTCCCAGATGAGCA	CAAGATGCACATCCGAAGCCA	Real-Time PCR for Snail
qSlug	CGAACTGGACACACATACAGTG	CTGAGGATCTCTGGTTGTGGT	Real-Time PCR for Slug
MSP-1-M	TTTAGTTTTCGGCG TTTTTTGTTTC	ACCTTTTCTCCCTAACCTTACCGAC	MSP PCR

(Continued)

name	Sense primer (5' to 3')	Anti-sense primer (5' to 3')	Application
MSP-1-U	TTTAGTTTTTGGTG TTTTTTGTTTTGT	ACCTTTTCTCCCTAACCTTACCAAC	MSP PCR
MSP-2-M	AATTATTAGGATTTTTTCGCGTCGT	TACGCTAATAATATACACCCGCGTC	MSP PCR
MSP-2-U	AATTATTAGGATTTTTTTGTGTTGT	AAACTACACTAATA ATATACACCCACATC	MSP PCR
ChIP-ctrl	CTGGGTGATGATAA GGATTAGTGAGATA	GGGAACAGATATTAG AAAGTCAGGCAAT	ChIP Real-Time PCR
ChIP-1	TGCTCTGAGTGCCTTGCGCATA	CTCTGTGCCTCCCTTTCCTCA	ChIP Real-Time PCR
ChIP-2	CACACCATCAGCGCAGCCT	CCACTCGCCCACCCATTCC	ChIP Real-Time PCR
ChIP-3	GGGACAGTCCGGGGGAGTTG	CCTGAGTGTTCGGGGCATCA	ChIP Real-Time PCR
ChIP-4	GAGTTCTCTCCCACCCCCACAC	ACACCCTTCAAACCAGCCACAT	ChIP Real-Time PCR
