# Supplementary material for: Downregulation of ATOH8 induced by EBV-encoded LMP1 contributes to the malignant phenotype of nasopharyngeal carcinoma
Source: Oncotarget. 2016 Mar 31;7(18):26765–79. doi: 10.18632/oncotarget.8503 (PMC5042013; doi:10.18632/oncotarget.8503)
Supplement: Supplementary file 2 [file oncotarget-07-26765-s002.docx]

|  |  | | | |  | | | |  | | | | |  | | |  | | | |  | | |  | | |  | | | |  | | |  | | | | |  |  |  |  |  |  |  |
| --- | --- | --- | --- | --- | --- | --- | --- | --- | --- | --- | --- | --- | --- | --- | --- | --- | --- | --- | --- | --- | --- | --- | --- | --- | --- | --- | --- | --- | --- | --- | --- | --- | --- | --- | --- | --- | --- | --- | --- | --- | --- | --- | --- | --- | --- |
| **Table S3. STR profiles of CNE1， HNE2 and C666 cells from different laboratories** | | | | | | | | | | | | | | | | | | | | | | | | | | | | | | | | | | | | | | | | | | | | | |
|  |  |  |  |  |  |  |  |  |  |  |  |  |  |  |  |  |  |  |  |  |  |  |  |  |  |  |  |  |  |  |  |  |  |  |  |  |  |  |  |  |  |  |  |  |  |
| Cell lines | |  | CNE-1 | | | |  | CNE-1 | | | |  | CNE-1 | | | | |  | CNE-1 | | | |  | HNE-2 | | | |  |  | HNE-2 | | | | |  |  |  | C666 | | | |  | C666 | | |
| Source | |  | Our lab | | | |  | K-W Lo's lab | | | |  | JHS | | | | |  | HKU | | | |  | Our lab | | | |  |  | K-W Lo's lab | | | | |  |  |  | Our lab | | | |  | K-W Lo's lab | | |
| Alleles | |  | 1 | 2 | | 3 |  | 1 | | 2 | 3 |  | 1 | | 2 | 3 | |  | 1 | 2 | | 3 |  | 1 | 2 | 3 | |  |  | 1 | | 2 | 3 | |  |  |  | 1 | | 2 | 3 |  | 1 | 2 | 3 |
| D5S818 | |  | 11 | 12 | |  |  | 11 | | 12 |  |  | 11 | | 12 |  | |  | 11 | 12 | |  |  | 11 | 12 |  | |  |  | 11 | | 12 |  | |  |  |  | 11 | | 12 |  |  | 11 | 12 |  |
| D21S11 | |  | 27 | 29 | |  |  | 27 | | 30 |  |  | 27 | | 30 |  | |  | 27 | 30 | |  |  | 27 | 30 |  | |  |  | 27 | | 30 |  | |  |  |  | 29 | | 31 |  |  | 29 | 31 |  |
| D18S51 | |  | 13 | 16 | |  |  | 13 | | 16 |  |  | 13 | | 16 |  | |  | 13 | 16 | |  |  | 13 | 16 |  | |  |  | 13 | | 16 |  | |  |  |  | 17 | | 17 |  |  | 17 | 17 |  |
| D3S1358 | |  | 15 | 18 | |  |  | 15 | | 18 |  |  | 15 | | 18 |  | |  | 15 | 18 | |  |  | 15 | 18 |  | |  |  | 15 | | 18 |  | |  |  |  | 16 | | 17 |  |  | 16 | 17 |  |
| D13S317 | |  | 10 | 12 | | 13 |  | 10 | | 12 | 13 |  | 10 | | 12 | 13.3 | |  | 10 | 12 | | 13.3 |  | 10 | 12 | 13.3 | |  |  | 10 | | 12 | 13.3 | |  |  |  | 8 | | 11 |  |  | 8 | 11 |  |
| D7S820 | |  | 10 | 12 | |  |  | 10 | | 12 |  |  | 10 | | 12 |  | |  | 10 | 12 | |  |  | 10 | 12 |  | |  |  | 10 | | 12 |  | |  |  |  | 10 | | 11 | 12 |  | 10 | 11 | 12 |
| D16S539 | |  | 9 | 10 | |  |  | 9 | | 10 |  |  | 9 | | 10 |  | |  | 9 | 10 | |  |  | 9 | 10 |  | |  |  | 9 | | 10 |  | |  |  |  | 10 | | 10 |  |  | 10 | 10 |  |
| CSF1PO | |  | 10 | 11 | |  |  | 10 | | 11 |  |  | 10 | | 11 |  | |  | 10 | 11 | |  |  | 10 | 11 |  | |  |  | 10 | | 11 |  | |  |  |  | 11 | | 15 | 16 |  | 11 | 15 | 16 |
| vWA | |  | 14 | 16 | |  |  | 14 | | 16 |  |  | 14 | | 16 |  | |  | 14 | 16 | |  |  | 14 | 16 |  | |  |  | 14 | | 16 |  | |  |  |  | 15 | | 17 | 18 |  | 15 | 17 | 18 |
| D8S1179 | |  | 12 | 16 | |  |  | 12 | | 16 |  |  | 12 | | 16 |  | |  | 12 | 16 | | 17 |  | 12 | 16 |  | |  |  | 12 | | 16 |  | |  |  |  | 13 | | 15 |  |  | 13 | 15 |  |
| TPOX | |  | 8 | 12 | |  |  | 8 | | 12 |  |  | 8 | | 12 |  | |  | 8 | 12 | |  |  | 8 | 12 |  | |  |  | 8 | | 12 |  | |  |  |  | 8 | | 11 |  |  | 8 | 11 |  |
| TH01 | |  | 6 | 7 | | 9 |  | 6 | | 7 | 9 |  | 6 | | 7 | 9 | |  | 6 | 7 | | 9 |  | 9 | 9 |  | |  |  | 9 | | 9 |  | |  |  |  | 6 | | 8 |  |  | 6 | 8 |  |
| FGA | |  | 18 | 21 | |  |  | 18 | | 21 |  |  | 18 | | 21 |  | |  | 18 | 21 | |  |  | 18 | 21 |  | |  |  | 18 | | 21 |  | |  |  |  | 21 | | 24 |  |  | 21 | 24 |  |
| PentaD | |  | 9 | 12 | |  |  | 9 | | 12 |  |  | 9 | | 12 |  | |  | 9 | 12 | |  |  | 9 | 12 |  | |  |  | 9 | | 12 |  | |  |  |  | 9 | | 10 |  |  | 9 | 10 |  |
| PentaE | |  | 15 | 17 | | 20 |  | 15 | | 17 | 20 |  | 15 | | 17 | 20 | |  | 15 | 17 | | 20 |  | 17 | 20 |  | |  |  | 17 | | 20 |  | |  |  |  | 11 | | 15 |  |  | 11 | 15 |  |
| D19S433 | |  | 13 | 13 | |  |  | 13 | | 13 |  |  | 13 | | 13 |  | |  | 13 | 13 | |  |  | 13 | 13 |  | |  |  | 13 | | 13 |  | |  |  |  | 12 | | 15 |  |  | 12 | 15 |  |
| D6S1043 | |  | 11 | 18 | |  |  | 11 | | 18 |  |  | 11 | | 18 |  | |  | 18 | 14 | |  |  | 11 | 14 | 18 | |  |  | 11 | | 14 | 18 | |  |  |  | 19 | | 19 |  |  | 19 | 19 |  |
| D12S391 | |  | 20 | 21 | |  |  | 20 | | 21 |  |  | 20 | | 21 |  | |  | 20 | 21 | |  |  | 20 | 21 |  | |  |  | 20 | | 21 |  | |  |  |  | 24 | | 25 |  |  | 24 | 25 |  |
| D2S1338 | |  | 17 | 23 | |  |  | 17 | | 23 |  |  | 17 | | 23 |  | |  | 17 | 23 | |  |  | 17 | 23 |  | |  |  | 17 | | 23 |  | |  |  |  | 16 | | 23 |  |  | 16 | 23 |  |
| Amelogenin | |  | X | X | |  |  | X | | X |  |  | X | | X |  | |  | X | X | |  |  | X | X |  | |  |  | X | | X |  | |  |  |  | X | | Y |  |  | X | Y |  |
| K-W Lo's lab, the lab of professor Kwok-Wai Lo in The Chinese University of Hong Kong; JHS, Johns Hopkins Singapore; HKU, The University of Hong Kong | | | | | | | | | | | | | | | | | | | | | | | | | | | | | | | | | | | | | | | | | | | | | |
|  |  |  |  |  |  |  |  |  |  |  |  |  |  |  |  |  |  |  |  |  |  |  |  |  |  |  |  |  |  |  |  |  |  |  |  |  |  |  |  |  |  |  |  |  |  |
